# Age Disparities Among Patients With Type 2 Diabetes and Associated Rates of Hospital Use and Diabetic Complications

**DOI:** 10.5888/pcd16.180681

**Published:** 2019-08-01

**Authors:** David C. Lee, Ta’Loria Young, Christian A. Koziatek, Christopher J. Shim, Marcela Osorio, Andrew J. Vinson, Joseph E. Ravenell, Stephen P. Wall

**Affiliations:** 1 Ronald O. Perelman Department of Emergency Medicine, New York University School of Medicine, New York, New York; 2 Department of Population Health, New York University School of Medicine, New York, New York; 3 Touro College of Osteopathic Medicine, New York, New York; 4 California Northstate University College of Medicine, Elk Grove, California

## Abstract

**Introduction:**

Although screening for diabetes is recommended at age 45, some populations may be at greater risk at earlier ages. Our objective was to quantify age disparities among patients with type 2 diabetes in New York City.

**Methods:**

Using all-payer hospital claims data for New York City, we performed a cross-sectional analysis of patients with type 2 diabetes identified from emergency department visits during the 5-year period 2011–2015. We estimated type 2 diabetes prevalence at each year of life, the age distribution of patients stratified by decade, and the average age of patients by sex, race/ethnicity, and geographic location.

**Results:**

We identified 576,306 unique patients with type 2 diabetes. These patients represented more than half of all people with type 2 diabetes in New York City. Patients in racial/ethnic minority groups were on average 5.5 to 8.4 years younger than non-Hispanic white patients. At age 45, type 2 diabetes prevalence was 10.9% among non-Hispanic black patients and 5.2% among non-Hispanic white patients. In our geospatial analyses, patients with type 2 diabetes were on average 6 years younger in hotspots of diabetes-related emergency department use and inpatient hospitalizations. The average age of patients with type 2 diabetes was also 1 to 2 years younger in hotspots of microvascular diabetic complications.

**Conclusion:**

We identified profound age disparities among patients with type 2 diabetes in racial/ethnic minority groups and in neighborhoods with poor health outcomes. The younger age of these patients may be due to earlier onset of diabetes and/or earlier death from diabetic complications. Our findings demonstrate the need for geographically targeted interventions that promote earlier diagnosis and better glycemic control.

**Summary**

**What is already known on this topic?**

Substantial racial, ethnic, and geographic disparities exist in diabetes prevalence, likely attributable to differences in the age of onset of diabetes and health outcomes among different subgroups.

**What is added by this report?**

In New York City, racial and ethnic minority populations have a higher prevalence of diabetes than nonminority populations at the recommended screening age of 45. The average age of patients with type 2 diabetes in some neighborhoods is more than a decade younger than in other neighborhoods.

**What are the implications for public health practice?**

These findings suggest the need to target efforts to prevent and diagnose diabetes in specific geographic areas.

## Introduction

Poor glycemic control, microvascular diabetic complications, and frequent diabetes-related hospital use have been shown to cluster in the same neighborhoods (1,2). Therefore, certain communities have poorer health outcomes, higher morbidity and mortality, and a higher proportion of the financial burden associated with diabetes. Many of these areas have a higher proportion of racial/ethnic minority residents, including non-Hispanic black and Hispanic residents (3). Their higher burden of diabetes is attributable in part to socioeconomic status, environmental influences, and health behaviors (4–7).

Diabetes prevention before onset and optimal management after diagnosis are critical to reduce these disparities (8,9). The American Diabetes Association recommends screening for type 2 diabetes starting at age 45 (10). The guidelines also suggest that earlier screening of persons at high risk may be warranted as a part of ongoing medical care (11). These recommendations are complicated in clinical practice because many people who are at high risk for diabetes also have limited access to medical care. In these populations, diagnosis may be delayed, which may have important implications for the development of long-term diabetic complications and early diabetes-related death (12).

The objective of this study was to investigate the age distribution of patients with type 2 diabetes in light of American Diabetes Association screening recommendations at age 45. Previous studies showed that claims data can be used to estimate the prevalence of diabetes and its associated complications and that claims data compare favorably with traditional health survey estimates (2,13). These studies demonstrated that the demographic distribution of unique emergency patients is similar to the demographic distribution of census estimates of the general population and is thus useful for tracking diabetes cross-sectionally for a large proportion of the population in a given geographic area (2).

In this study, we used all-payer claims data to analyze the age of patients with type 2 diabetes in New York City and to stratify patients by sex, race, and ethnicity. We also examined the average age of patients with type 2 diabetes living in previously identified “hotspots” (ie, geospatial clusters) of diabetes-related hospital use. These rates of hospital use are important because diabetes-related emergency department visits and inpatient hospitalizations account for nearly half of the large financial burden associated with diabetes (14). We also analyzed the average age of patients with type 2 diabetes in previously identified hotspots of macrovascular (eg, myocardial infarction and stroke) versus microvascular (eg, end-stage renal disease and non-traumatic lower extremity amputations) diabetic complications because they have significant consequences for patient outcomes (15).

## Methods

### Study design

Nearly 800,000 people in New York City have type 2 diabetes (16). To study the age distribution of these patients, we first assessed the prevalence of type 2 diabetes among patients in New York City who had at least 1 emergency department visit from 2011 through 2015 (2). We then calculated both the average age of patients with type 2 diabetes and the proportion of these patients in 10-year age groups (ie, 10–19, 20–29, 30–39, and so on). We then stratified these measures by sex, race, and ethnicity. We used geographic analysis to identify hotspots of both diabetes-related hospital use and diabetic complications, and then we examined the age of patients with type 2 diabetes living in these areas (17).

### Data sources

We obtained data on emergency department visits and inpatient hospitalizations from the Statewide Planning and Research Cooperative System (SPARCS), which is an all-payer claims database administered by the New York State Department of Health (18). We used these data to analyze the age of patients with type 2 diabetes and calculate the rate of diabetes-related emergency department visits and inpatient hospitalizations. We calculated these hospital use rates per resident by using tract-level 5-year estimates from the US Census Bureau’s American Community Survey 2011–2015 (19). We used SPARCS data also to estimate the prevalence of diabetic complications among patients with type 2 diabetes (1).

### Main outcome

Our main outcome was the age of patients with type 2 diabetes, which we assessed among unique patients who had at least 1 emergency department visit from 2011 through 2015. For patients with more than 1 emergency department visit during the 5-year period, we selected the age at a randomly selected visit. We used unique identifiers from the SPARCS database to account for multiple visits to different hospitals by the same person. We included patients aged 10 to 100 who had a home address that geocoded to a census tract in New York City. We then analyzed age as an average and also by the proportion of patients in each 10-year age group.

### Associated health outcomes

To analyze associations between our main outcome and key diabetes-related health measures, we analyzed the average age of patients with type 2 diabetes in geographic areas with a high prevalence of diabetic complications. We identified the proportion of patients with type 2 diabetes who also had a concurrent diagnosis of myocardial infarction, ischemic stroke, end-stage renal disease, or non-traumatic lower extremity amputation (Box) (1). We calculated the estimated prevalence of these conditions among unique patients who visited an emergency department at least once during the study period and had a primary or secondary diagnosis of type 2 diabetes during any of their previous emergency department visits.

**Box. ICD-9-CM and ICD-10-CM Codes for Type 2 Diabetes and Severe Diabetic Complications^a^**

**Table:** 

Diagnosis	ICD-9-CM Code	ICD-10-CM Code
Type 2 diabetes	250.x1, 250.x3	E11
Myocardial infarction	410, 412	I21, I22, I23, I25.2
Ischemic stroke	434, 436	I63, I66
End-stage renal disease	585.6	N18.6
Nontraumatic lower extremity amputations	V49.7, (p)84.1 (exclude 895, 896, 897)	Z89.4, Z89.5, Z89.6, (p)0Y6 (exclude S78, S88, S98)

Abbreviation: CM, Clinical Modification; ICD, International Classification of Diseases.

^a ^ICD-9-CM and ICD-10-CM are the official systems of assigning codes to diagnoses and procedures associated with hospital use in the United States (20,21).

We also analyzed measures of diabetes-related hospital use, which has been estimated to represent nearly half of the direct financial costs associated with diabetes and geographically overlaps with areas of poor glycemic control as measured by hemoglobin A_1c_ (14,22). Here, we calculated the per capita rates of diabetes-specific emergency department visits and inpatient hospitalizations separately. To create these 2 measures, we determined the average annual number of emergency department visits or inpatient hospitalizations with a primary (not secondary) diagnosis for type 2 diabetes, then divided that figure by the total number of residents at all ages as estimated by the American Community Survey 2011–2015 for each census tract (19).

### Statistical analysis

We analyzed the average age and prevalence of patients with type 2 diabetes by using standard descriptive statistics. We also mapped the average age of patients with type 2 diabetes by census tract and grouped the range of average ages into deciles. To identify geographic hotspots and coldspots of diabetic complications and diabetes-related health care use, we used the Getis-Ord Gi* statistic to find significant geographic clustering (23). We used the adjacent neighboring census tracts that shared a border (first-order queen contiguity) to identify local clusters where our estimates of diabetic complications and diabetes-related health care use were higher or lower than would be expected by chance. This analysis was row-standardized to account for variation in the number of neighboring census tracts across New York City. We then used a *t* test to compare the average age of patients with type 2 diabetes among census tracts identified as hotspots with the average of patients with type 2 diabetes among census tracts identified as coldspots for the diabetes-related health outcomes of interest.

In our analyses, we excluded 57 of 2,168 census tracts (2.6%) with a total population estimate of less than 100 people to limit the bias of areas with low population counts (mostly parks and airports). We also excluded 28 of the 2,168 census tracts (1.3%) with fewer than 30 patients that were identified as having type 2 diabetes to limit the bias of areas with an insufficient number of observations to provide an average age across patients with diabetes (1). To account for multiple comparisons among the 4 measures of diabetic complications and the 2 measures of diabetes-related health care use, we used a Bonferroni correction to adjust our α level, which corresponded to a *P* value of .008.

We performed statistical analyses in Stata 14.2 (StataCorp LLC). We created maps by using ArcGIS Desktop 10.3.1 (Esri), and we used GeoDa 1.8.14 (Center for Spatial Data Science at the University of Chicago) for geospatial analyses. Our study protocol was approved by the institutional review board at the New York University School of Medicine.

## Results

We identified 576,306 unique patients aged 10 to 100 who had visited an emergency department at least once from 2011 through 2015 and had a diagnosis of type 2 diabetes. Thus, our population sample identified more than half of people with type 2 diabetes in New York City. The age and sex distributions of our study population were similar to the distributions of the population in New York City as described by census data. However, the proportion of people who were non-Hispanic black, publicly insured, or uninsured was higher among unique emergency department patients than among the population in New York City (Table).

**Table:** Demographic Characteristics of New York City Population,^a^ Unique Emergency Department Patients,^b^ and Emergency Department Patients With Type 2 Diabetes

Characteristic	**Census Estimates, % (N = 7,388,165)**	Unique Emergency Department Patients, % (N = 5,556,650)	Emergency Department Patients With Type 2 Diabetes, % (N = 576,306)
**Age group, y**			
10–19	12.9	13.4	0.5
20–29	19.0	21.6	2.2
30–39	17.8	17.6	5.1
40–49	15.4	14.1	12.4
50–59	14.4	13.0	22.5
60–69	10.7	9.1	23.5
70–79	5.9	5.9	18.8
≥80	4.0	5.3	15.0
**Sex**			
Male	47.6	45.1	45.4
Female	52.4	54.9	54.6
**Race/ethnicity**			
Non-Hispanic white	32.5	22.6	20.2
Non-Hispanic black	22.4	28.9	32.8
Hispanic	28.9	26.4	24.8
Asian	13.4	6.0	6.6
Other	2.8	16.1	15.6
**Health insurance**			
Private	47.2	27.1	15.7
Medicare	12.0	13.7	41.0
Medicaid	24.1	36.5	32.1
Self-pay	12.4	18.4	8.3
Other	4.3	4.3	2.9

a Data source: American Community Survey 2011–2015 (19).

b Data source: New York State Department of Health Statewide Planning and Research Cooperative System (18).

### Estimated prevalence at specific ages by sex, race, and ethnicity

**Figure 1:**
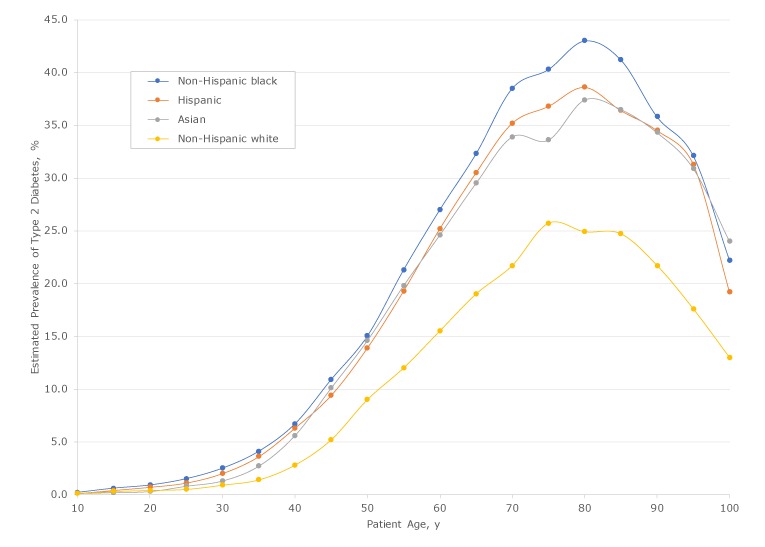
Estimated prevalence and age distribution of patients with type 2 diabetes by race/ethnicity among 576,306 unique patients aged 10 to 100 years who had visited an emergency department at least once from 2011 through 2015 in New York City. Data source: New York State Department of Health Statewide Planning and Research Cooperative System (18).

The estimated prevalence of type 2 diabetes increased with age from 0.1% among emergency department patients aged 10 to 34.2% among patients aged 78. Peak prevalence was higher among non-Hispanic black patients, at 43.0%, than among non-Hispanic white patients, at 25.7%. Among all emergency department patients aged 25 or older, the prevalence of type 2 diabetes was higher among racial/ethnic minority patients than among non-Hispanic white patients of the same age (Figure 1). Differences by sex were not large: male patients had a 1- to 2-percentage point higher prevalence of type 2 diabetes than female patients between the ages of 40 and 75.

**Table:** 

**Age**	Non-Hispanic Black, %	Hispanic, %	Asian, %	Non-Hispanic White, %
10	0.2	0.1	0.1	0.1
15	0.6	0.4	0.2	0.3
20	0.9	0.7	0.3	0.4
25	1.5	1.1	0.8	0.5
30	2.5	2.0	1.3	0.9
35	4.1	3.6	2.7	1.4
40	6.7	6.3	5.6	2.8
45	10.9	9.4	10.1	5.2
50	15.3	13.9	14.6	9.0
55	21.3	19.3	19.8	12.0
60	27.0	25.1	24.6	15.5
65	32.3	30.5	29.5	19.0
70	38.5	35.2	33.9	21.7
75	40.3	36.8	33.6	25.7
80	43.0	38.6	37.4	24.9
85	41.2	36.4	36.5	24.7
90	35.8	34.5	34.3	21.7
95	32.1	31.3	30.9	17.6
100	22.2	19.2	24.0	13.0

The estimated prevalence of type 2 diabetes among non-Hispanic white patients aged 45 was 5.2%. Among non-Hispanic black patients aged 45, the prevalence of type 2 diabetes was 10.9%, more than twice as high as that for non-Hispanic white patients. Among non-Hispanic black patients, the prevalence of type 2 diabetes reached 5.2% by age 37 or 38. In addition, 9.4% of Hispanic patients and 10.1% of Asian patients had diabetes at age 45. The proportion of patients with type 2 diabetes who were younger than 45 was 6.5% among non-Hispanic white patients, 14.0% among non-Hispanic black patients, 15.7% among Hispanic patients, and 10.4% among Asian patients.

### Age distribution by sex, race, and ethnicity

The average age of male patients with type 2 diabetes was 2.6 years younger than the average age of female patients. This difference was most pronounced among non-Hispanic white patients, at 4.4 years, and the least pronounced among Asian patients, at 1.5 years. The average age of non-Hispanic white patients with type 2 diabetes was 68.8. Non-Hispanic black patients were on average 8.3 years younger, Hispanic patients were 8.5 years younger, and Asian patients were 5.5 years younger.

**Figure 2:**
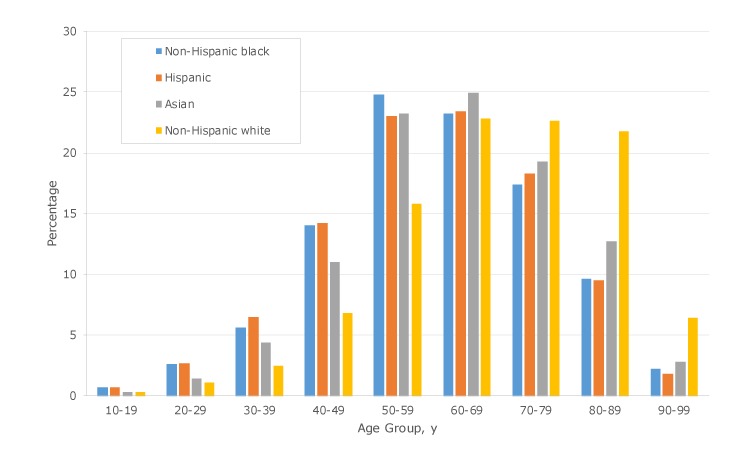
Age distribution by sex, race, and ethnicity among patients with type 2 diabetes among 576,306 unique patients aged 10 to 100 years who had visited an emergency department at least once from 2011 through 2015 in New York City. Data source: New York State Department of Health Statewide Planning and Research Cooperative System (18).

Analyzing the age distribution by decades, 22.8% of non-Hispanic white patients with type 2 diabetes were aged 60 to 69, followed closely by non-Hispanic white patients aged 70 to 79, at 22.6%. In contrast, the highest proportion of non-Hispanic black patients with type 2 diabetes was aged 50 to 59. They represented 24.8% of all non-Hispanic black patients with type 2 diabetes, followed closely by those aged 60 to 69, at 23.2%. However, we found steep declines in subsequent decades. Only 17.4% of non-Hispanic black patients with type 2 diabetes were aged 70 to 79, and only 9.6% were aged 80 to 89. We observed similar trends among Hispanic and Asian patients, although declines were slightly less steep among Asian patients (Figure 2).

**Table:** 

Age Group	Non-Hispanic Black, %	Hispanic, %	Asian, %	Non-Hispanic White, %
10-19	0.7	0.7	0.3	0.3
20-29	2.6	2.7	1.4	1.1
30-39	5.6	6.5	4.4	2.5
40-49	14.0	14.2	11.0	6.8
50-59	24.8	23.0	23.2	15.8
60-69	23.2	23.4	24.9	22.8
70-79	17.4	18.3	19.3	22.6
80-89	9.6	9.5	12.7	21.8
90-99	2.2	1.8	2.8	6.4

### Geographic distribution of average age

**Figure 3:**
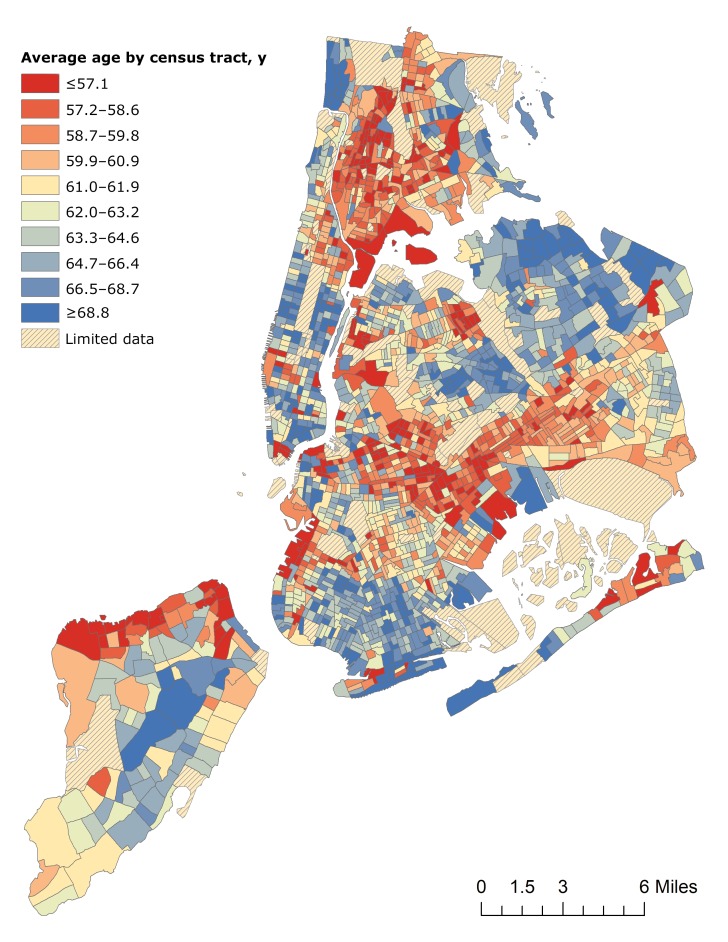
Average age of patients with type 2 diabetes, by census tract, among 576,306 unique patients aged 10 to 100 years who had visited an emergency department at least once from 2011 through 2015 in New York City. Data source: New York State Department of Health Statewide Planning and Research Cooperative System (18).

We found an almost 12-year difference in the average age of patients with type 2 diabetes between the census tracts at the 10th and 90th percentiles. Excluding census tracts with low population counts, the lowest decile had an average age of 57.1 or younger (Figure 3). The highest decile had an average age of 68.8 or older. 

### Average age and associated health outcomes

Among the 2,083 census tracts analyzed in our study, we identified several geographic clusters of diabetes-related hospital use and diabetic complications. Patients with type 2 diabetes in census tracts identified as hotspots of diabetes-related emergency department use were an average of 5.9 years younger than patients in coldspots (*P* < .001). Similarly, patients with type 2 diabetes were an average of 5.8 years younger in hotspots of diabetes-related inpatient hospitalizations than patients in coldspots (*P* < .001).

For microvascular diabetic complications, patients with type 2 diabetes in hotspots of end-stage renal disease were 2.2 years younger than patients in coldspots (*P* < .001). Patients with type 2 diabetes in hotspots of non-traumatic lower extremity amputations were 1.5 years younger than patients in coldspots (*P* = .002). For macrovascular complications of diabetes, the age trend was reversed. Patients with type 2 diabetes in hotspots of myocardial infarction were 5.7 years older than patients in coldspots (*P* < .001). Similarly, patients with type 2 diabetes in hotspots of ischemic stroke were 3.8 years older than patients in coldspots of these complications.

## Discussion

Our study analyzed the age distribution of patients with type 2 diabetes in New York City and found that the average age of these patients was younger in hotspots of diabetes-related emergency department visits and inpatient hospitalizations. We further found that the average age of patients with type 2 diabetes was younger in areas identified as hotspots of microvascular (but not macrovascular) diabetic complications. We observed substantial disparities in the age of patients with type 2 diabetes when stratified by sex, race, and ethnicity. These findings suggest that the age of patients with type 2 diabetes may play a role in the high costs, morbidity, and mortality associated with type 2 diabetes.

Previous studies demonstrated that an earlier age of onset results in a higher risk of diabetic complications (24,25). Data obtained in a study linking duration of diagnosis with diabetes-related outcomes found an interaction between the duration of diabetes and the risk of microvascular events, but not macrovascular events (26). The study also found that this effect was greatest at a younger rather than older age. Our own study found a similar pattern, whereby microvascular diabetic complications were associated with a younger average age of patients with type 2 diabetes. In contrast, macrovascular diabetic complications were associated with an older average age.

These findings may speak to distinct subpopulations of patients with type 2 diabetes that are demographically different and geographically separate and face disparate clinical trajectories of long-term diabetic complications. Other studies also established a link between higher rates of microvascular complications and the level of glycemic control when measuring HbA_1c_ (27). The geographic distribution of younger average age among patients with type 2 diabetes in our study was similar to the geographic distribution in a recently published map of poor glycemic control in New York City (21). These wide disparities in diabetes burden may be largely driven by a subpopulation of patients who have an earlier age of onset, have poorer glycemic control, experience more microvascular complications than macrovascular complications, and die at a much earlier age.

In our study, the distribution of patients with type 2 diabetes across age decades varied substantially by race and ethnicity. The highest proportion (not prevalence) of non-Hispanic black patients with type 2 diabetes were in their 50s: almost one-quarter (24.8%) were in their 50s, nearly a decade younger than the highest proportion (22.8%) of non-Hispanic white patients, who were in their 60s. Several factors might explain these disparities in age distribution. First, as our prevalence estimates demonstrate, racial/ethnic minority patients develop type 2 diabetes at a younger age than non-racial/ethnic minority patients. Second, racial/ethnic minority patients with type 2 diabetes might be dying earlier of either diabetic complications or other causes. Although diabetes prevalence is still high among racial/ethnic minority patients at older ages, our most startling finding was the low proportion of racial/ethnic minority patients with type 2 diabetes who were alive in their 80s and 90s.

The current standards for type 2 diabetes screening do not recommend community-based screening (28). The concerns cited in these recommendations are that people identified through community-based screening as having diabetes may not seek or have access to appropriate follow-up and that community-based testing may be poorly targeted (10,29). The challenge in these recommendations is that the populations at highest risk of type 2 diabetes are often the same groups that do not have a regular source of medical care (30). Our maps of New York City identified areas with the youngest average age of patients with type 2 diabetes, which are some of the same neighborhoods where people report a lack of medical care (31). These communities may be affected by cultural health behaviors or local conditions (eg, poor food environment) that worsen these disparities, but these behaviors and conditions might also provide opportunities for intervention (32). Data on residential addresses might enable identification of specific areas (eg, block or block groups) where interventions are needed most.

Our study has several limitations. The fidelity of certain variables in the claims data can vary by hospital (eg, by ownership, level of care, or specialty designations). Certain hospitals are less reliable than others at coding patients as having diabetes. However, these institutions tend to be public hospitals that provide care for a higher proportion of racial/ethnic minority patients; thus, this unreliability would bias our study toward the null. In addition, certain hospitals have historically been inaccurate in coding race and ethnicity categories, often coding “other” for these variables. This analysis is limited to New York City, which is racially and ethnically diverse and a dense urban environment; thus, our findings may not generalize to other areas. Finally, many parts of New York City are on islands, and edge effects may have affected our geospatial results.

If patients with high diabetes-related health care expenditures, morbidity, and mortality develop diabetes at an early age and are unlikely to obtain medical care, then the public health approach to screening for type 2 diabetes may need to be revised. Given clustering of poor outcomes in certain neighborhoods, community-based diabetes screening may be an approach that increases awareness among groups unlikely to access medical care and would expand diagnosis outside of clinical settings (33). Doing so, we may be able to develop and deliver high-yield and culturally relevant interventions that can reduce these disparities.
